# Adults’ Acceptance of COVID-19 Vaccine for Children in Selected Lower- and Middle-Income Countries

**DOI:** 10.3390/vaccines10010011

**Published:** 2021-12-22

**Authors:** Suzanna Awang Bono, Ching Sin Siau, Won Sun Chen, Wah Yun Low, Edlaine Faria de Moura Villela, Supa Pengpid, M Tasdik Hasan, Philippe Sessou, John D. Ditekemena, Bob Omoda Amodan, Mina C. Hosseinipour, Housseini Dolo, Joseph Nelson Siewe Fodjo, Robert Colebunders

**Affiliations:** 1 School of Social Science, Universiti Sains Malaysia, Gelugor 11800, Malaysia; suzanna.bono@usm.my; 2 Centre for Community Health Studies (ReaCH), Faculty of Health Sciences, Universiti Kebangsaan Malaysia, Kuala Lumpur 50300, Malaysia; 3 Department of Health Science and Biostatistics, Faculty of Health, Arts and Design, Swinburne University of Technology, Hawthorn, VIC 3122, Australia; wchen@swin.edu.au; 4 Faculty of Medicine, Universiti Malaya, Kuala Lumpur 50603, Malaysia; lowwy@um.edu.my; 5 Asia Europe Institute, Universiti Malaya, Kuala Lumpur 50603, Malaysia; 6 Disease Control Coordination, Sao Paulo State Health Department, São Paulo 01246-000, Brazil; edlaine@alumni.usp.br; 7 Institute of Tropical Pathology and Public Health, Federal University of Goiás, Goiania 74690-900, Brazil; 8 ASEAN Institute for Health Development, Mahidol University, Nakhon Pathom 73170, Thailand; supa.pen@mahidol.ac.th; 9 Jeeon Bangladesh Ltd., Dhaka 1207, Bangladesh; tasdikhdip@yahoo.com; 10 Department of Primary Care & Mental Health, University of Liverpool, Liverpool L69 3BX, UK; 11 Research Unit on Communicable Diseases, Polytechnic School of Abomey-Calavi, University of Abomey-Calavi, Cotonou 01 BP 526, Benin; sessouphilippe@yahoo.fr; 12 Kinshasa School of Public Health, University of Kinshasa, Kinshasa 7948, Democratic Republic of the Congo; jditekemena@hotmail.fr; 13 Uganda Public Health Fellowship Program, Loudel Towers, Level 4, Kampala 7272, Uganda; bomoda@musph.ac.ug; 14 School of Medicine, University of North Carolina at Chapel Hill, Chapel Hill, NC 27599, USA; mina_hosseinipour@med.unc.edu; 15 University of North Carolina Malawi, Lilongwe 999119, Malawi; 16 International Center of Excellence in Research, Faculty of Medicine and Odonto Stomatology, University of Sciences, Techniques and Technology of Bamako, Bamako BP 1805, Mali; dolohousseini@yahoo.fr; 17 Global Health Institute, University of Antwerp, 2000 Antwerp, Belgium; josephnelson.siewefodjo@uantwerpen.be (J.N.S.F.); robert.colebunders@uantwerpen.be (R.C.); 18 Brain Research Africa Initiative (BRAIN), Yaounde P.O. Box 25625, Cameroon

**Keywords:** COVID-19 vaccination, children, lower- and middle-income countries, parents, caretaker, Brazil, Malaysia, Thailand, Bangladesh, Africa

## Abstract

Since emergency approval of COVID-19 vaccines for children aged between 12 and 15 years old was recently obtained in the United States and Europe, we aimed to assess the willingness to vaccinate children with a COVID-19 vaccine in lower- and middle-income countries (LMICs). Therefore, we launched an online cross-sectional survey in several LMICs. Questions relating to socio-demographic information, knowledge of COVID-19, level of fear/worry of being infected with COVID-19, and willingness to vaccinate children with the COVID-19 vaccine at 50%, 75% and 95% effectiveness levels, were asked. Of the 6571 participants (mean age = 39 ± 14 years), 64.0%, 72.6%, and 92.9% were willing to vaccinate children at 50%, 75%, and 95% effectiveness levels, respectively. Respondents who were undergraduates, who were more worried/fearful about COVID-19, had higher knowledge scores regarding COVID-19, and a higher belief that COVID-19 vaccination is important to protect others, were more willing to accept COVID-19 vaccination of children. COVID-19 vaccination of children will limit the spread of the virus, especially in schools; it may decrease the need for school closures which has a negative effect on child development. Findings from this study are useful for health promotion strategies during COVID-19 vaccination implementation among children in LMICs.

## 1. Introduction

The coronavirus disease 2019 (COVID-19) was declared a pandemic by the World Health Organization (WHO) on 11 March 2020 and precipitated a global health emergency [1]. As of 9 December 2021, a total of 265,713,467 confirmed cases and 5,260,888 deaths were reported [2]. Against the unmitigated increase of new cases, the rapid authorization and roll out of COVID-19 vaccines serves as an important prevention measure against COVID-19 infection. COVID-19 vaccination is very pertinent to any government’s strategy in recovery and achieving herd immunity. Vaccines play a critical role in preventing death and hospitalization caused by infectious diseases [3,4]. Vaccine trials have reported encouraging results indicating that a COVID-19 vaccine is safe and produces a good immune response [5,6]. The Pfizer BioNTech and Moderna vaccines were authorized for emergency use by the Food and Drug Administration in the United States of America in December 2020 for individuals who were 16 years old and older [3]. In the same month, the first COVID-19 vaccine, Pfizer BioNTech, also received emergency authorization in the European Union [7]. COVID-19 vaccines have been shown to be effective in controlling the spread of the disease [4]. Further awareness of the need for vaccination and additional protective behaviors is required to control the pandemic. Among lower-income countries, the COVAX initiative was implemented to enable equitable and accelerated distribution of the vaccines.

As of 7 December 2021, a total of 3.53 billion individuals were fully vaccinated [8]. As the COVID-19 vaccine is being rolled out globally and an increasing number of the adult population is being vaccinated, more interest has been generated in extending COVID-19 vaccination to children. In May 2021, Canada and the United States of America granted emergency approval of the COVID-19 vaccine for use among children aged between 12 and 15 years [9,10].

The success of a vaccination program depends on rates of uptake among the population. Adverse events, especially side effects may have led some people to express concerns about getting vaccinated, delay getting vaccinated or even strongly oppose vaccination, and may have reduced their confidence in national safety monitoring systems. Further, a challenge in communicating the importance of COVID-19 vaccination is that younger adults are typically less clinically affected by COVID-19 infection and so may see limited value in getting vaccinated [11]. In addition, misinformation about future COVID-19 vaccines has circulated on social media platforms [12], further amplified by already high levels of vaccine misinformation in general [13]. Vaccine hesitancy overall has risen so substantially that the WHO now considers it a major threat to global health. A specific challenge for a COVID-19 vaccine is that its expedited development may contribute to the public impression that the vaccine will not have been sufficiently tested for safety and efficacy [13,14,15].

Several factors have led to hesitancy among individuals and governments to start vaccinating children. Firstly, COVID-19 symptoms are milder and rarely lead to hospitalization and mortality among children, compared to adults [16]. In view of this, especially since only approximately 44.7% of the global population was fully vaccinated as of 7 December 2021, there is a greater urgency to vaccinate adults [8]. In addition, the safety of COVID-19 vaccines for administration among children is still being established [17].

Compared to a very large number of studies addressing COVID-19 vaccine acceptance among adults [18], there is a limited number of studies examining COVID-19 vaccine acceptance for children especially among low- and middle-income countries (LMICs) [19,20,21]. Given the feasibility of vaccinating children in LMICs with the COVID-19 vaccine in the near future, our research questions were: (1) What are the COVID-19 vaccine acceptance rates for children at 50%, 75%, and 95% effectiveness levels, and (2) What are the factors affecting vaccine acceptance for children? In an online survey in three South East Asian countries, Brazil, and several African countries, we investigated willingness to vaccinate children with vaccines of three different levels of effectiveness, 50%, 75%, and 95%. The 95% level was chosen based on the initial results of the COVID-19 messenger RNA (mRNA) vaccines showing an effectiveness of 95% [22], the 50% level is the minimum level for a vaccine to be approved [23], and the 75% level is an intermediate level where vaccination could be considered depending on severity of the disease [24,25]. We hypothesized that the higher the vaccine effectiveness, the higher the vaccine acceptance would be. We also explored the factors that may affect vaccine acceptance for children.

## 2. Materials and Methods

### 2.1. Design and Participants

This was a descriptive cross-sectional study. This study is a part of the International Citizen Project (ICP) COVID-19 (https://www.icpcovid.com/en/form/covid-19-vaccine-survey, accessed on 4 November 2021) whereby a series of large-scale online surveys are developed to understand country-level adherence to the interventions recommended by the WHO. The English version of the questionnaire was pilot-tested among ICPCovid team members and translated into the main national languages of the participating countries. We targeted adult members of the general public to understand their attitude toward COVID-19 vaccination of children. Participants were recruited based on specific inclusion criteria which were: (1) being at least 18 years old, and (2) providing informed consent to participate in this study. Participants were recruited in LMICs namely, Brazil, Malaysia, Thailand, Bangladesh, and African countries, including the Democratic Republic of Congo, Uganda, and Malawi. Ethical approval for this study was granted by the ethics committees in respective participating countries (see author statements for more details).

### 2.2. Materials

There were three parts to the questionnaire. In the first part of the questionnaire, demographic information (e.g., age, sex, country of residence, educational level, studying or working in healthcare, number of people currently living with according to age, self-perceived socio-economic status, self-perceived area of residence, and working/studying from home) was collected. These variables were chosen as they had been shown in past studies to be associated with vaccine acceptance [26,27]. Those who are working or studying from home may be less open to vaccinate children since they are less at risk for contracting the virus in comparison with individuals who are not working or studying from home. In the second part, participants were asked about health status, knowledge of COVID-19, and their level of fear/worry of being infected with COVID-19. With respect to the COVID-19 knowledge items, each “Yes” answer scored 1 point, and each “No” answer scored 0 point. These items were: (1) possibility of being re-infected after recovering from a previous COVID-19 infection, (2) COVID-19 infection could be prevented by a vaccine, and (3) there is currently an effective vaccine against COVID-19. The third and last part of the questionnaire consisted of three questions regarding the willingness of participants to vaccinate children with the COVID-19 vaccine at 50%, 75% and 95% effectiveness levels.

### 2.3. Procedure

The original questionnaire was in English. The research collaborators from the participating countries translated the questionnaire into their own national language. The translated questionnaires were pilot-tested among the team members of the ICPCovid consortium belonging to the respective countries. Self-administered online questionnaires were disseminated through social media platforms, such as WhatsApp, Facebook, Messenger, Twitter, and university webpage portals, between 10 December 2020 and 9 February 2021, by the researchers to their personal and professional networks. Participation was voluntary. Participants were asked to provide informed consent before attempting the questionnaire. Participants’ information and answers were kept private and confidential by not retrieving any information that could lead the investigators to the participants.

### 2.4. Weighting

Prior to conducting data analysis, all data were weighted. The weighting process was previously described in detail by Bono et al. [28].

### 2.5. Statistical Analysis

Descriptive statistics were presented as means and standard deviations (SD) for continuous variables, and categorical variables were summarized using frequency and percentage. Chi-square tests were used to test the associations between the demographic characteristics and vaccine acceptance at 50%, 75%, and 95% effectiveness levels. Independent samples t-tests were used to test whether there were significant differences in vaccine acceptance at all levels in terms of worry/fearfulness of being infected by COVID-19, total knowledge score, and importance of getting the vaccine to protect others. A series of multiple logistic regression analyses was conducted with vaccine acceptance as the dependent variable, at 50%, 75%, and 95% effectiveness levels. The dependent variable was coded 1 = yes and 0 = no/no opinion/not applicable. We verified all assumptions for multiple logistic regression and used the Spearman’s Rank correlation coefficient and Pearson’s correlation coefficient to investigate the multi-collinearity of the factors. The variable “Importance of taking COVID-19 vaccine to protect self” was excluded from the regression analyses due to its high correlation with “Importance of taking COVID-19 vaccine to protect others”. A p-value of less than 0.05 (two-tailed) was deemed statistically significant. All analyses were conducted using SPSS [29].

## 3. Results

A total of 10,183 individuals responded to the questionnaire, of which 6571 (64.5%; mean age = 39 ± 14) responded to the question regarding the willingness to vaccinate children. Post-hoc power analysis was conducted using logistic regression in G*Power (version 3.1.2) [30]. Based on the 95% effectiveness logistic regression model, with *n* = 6571, the odds ratio for females in predicting vaccine acceptance for children = 0.55, R2 of other X = 0.48, the critical z = −1.96 and power (1-β err prob) = 1.00. Therefore, this study was adequately powered. Of the participants (*n* = 6571), the majority were female (64.8%), had postgraduate education (50.3%), belonged to the upper-middle income category (46.0%), lived in an urban setting (83.6%), and worked/studied from home (58.4%) (Table 1). All countries, except the African countries, had more female participants. About one third (29.9%) were students/workers in the healthcare sector. In terms of household composition, 30.5% reported living with children < 12 years old, whilst 20.5% reported living with children 12 to 17 years old. Since the beginning of the COVID-19 outbreak, 8.8% had tested positive for COVID-19 and 36.2% had tested COVID-19 negative at the time of the survey.

Regarding the participants’ willingness to vaccinate children, 64.0% (95%CI [62.7, 65.2]), 72.6% (95%CI [71.5, 73.7]), and 92.9% (95%CI [92.2, 93.5]) were willing to vaccinate at the 50%, 75%, and 95% effectiveness levels, respectively. However, only 66.4% of the participants believed that there is currently an effective vaccine against COVID-19. Most of the participants reported that it is extremely important to take the COVID-19 vaccine to protect others (72.0%) (Table 2). Chi-square and independent samples *t*-tests revealed significant findings for all variables except residential setting, with vaccine acceptance at 95% effectiveness level, and healthcare worker/student status, with vaccine acceptance at 75% and 95% effectiveness levels (Table 3).

Compared to Brazil, all other countries reported lower willingness to vaccinate children, with Thailand having the lowest level of acceptance at 50% effectiveness (aOR: 0.06, 95%CI [0.05, 0.08], *p* < 0.001) and 75% effectiveness (aOR: 0.06, 95%CI [0.04, 0.08], *p* < 0.001) (Table 3). However, at 95% effectiveness level, child vaccine acceptance was only significantly lower in Malaysia (aOR: 0.58, 95%CI [0.38, 0.88], *p* = 0.011) and the African countries (aOR: 0.23, 95%CI [0.16, 0.32], *p* < 0.001), compared to Brazil.

At 50% and 75% effectiveness, individuals aged 30–39 years old were least likely to accept vaccination of children but were most likely to accept the vaccination in cases of an effectiveness level of 95%. Females were more in favor of child vaccination than males at 75% effectiveness (aOR: 1.17, 95%CI [1.01, 1.35], *p* = 0.032). However, at 95% effectiveness, females were less in favor of child vaccination compared to males (aOR: 0.54, 95%CI [0.44, 0.73], *p* < 0.001). At 50% vaccine effectiveness, individuals who lived with children 12 to 17 years old had higher acceptance rates for the vaccination of children (aOR: 1.27, 95%CI [1.07, 1.50], *p* = 0.006), while at 95% effectiveness level, those who lived with children < 12 years old were more likely to accept child vaccination (aOR: 1.30, 95%CI [1.04, 1.63], *p* = 0.023). Those with an undergraduate degree were more willing to vaccinate children compared to individuals with primary/secondary education, with the highest odds ratio at 95% effectiveness (aOR: 2.33, 95%CI [1.71, 3.19], *p* < 0.001).

At 95% effectiveness, individuals with upper-middle income had higher odds of accepting vaccination for children (aOR: 1.77, 95%CI [1.04, 3.00], *p* = 0.034) whilst those living in urban area (aOR: 0.47, 95%CI [0.28, 0.79], *p* = 0.0.5) had lower odds of vaccine acceptance for children.

At 50% and 75% effectiveness levels, participants who had tested positive for COVID-19 were less likely to accept vaccination of children (aOR: 0.78, 95%CI [0.61, 0.99], *p* = 0.043 and aOR: 0.76, 95%CI [0.60, 0.97], *p* = 0.027, respectively).

Across all effectiveness levels, individuals who were more worried/fearful about COVID-19, who had higher knowledge about COVID-19, and who believed that taking the COVID-19 vaccine is important to protect others, were more likely to accept COVID-19 vaccination of children (Table 4).

## 4. Discussion

With the increasing availability of COVID-19 vaccines worldwide, understanding the factors influencing parents’ or caregivers’ willingness to vaccinate their children is critically important for vaccine policy decisions. In this international online survey 92.9% of the participants were willing to vaccinate children at 95% vaccine effectiveness, which is high in comparison with other studies. In an international cross-sectional survey of emergency departments in six countries, 65% of caregivers reported that they intended to vaccinate their child against COVID-19, once a vaccine was available [20]. In a study among factory workers in Shenzhen, China, 72.6% of parents would accept COVID-19 vaccination of their children [31]. In Zambia, in a nested study within a measles-rubella mass vaccination campaign, 92% of the parents reported that they intended to have their child vaccinated against COVID-19 [19]. Further, in the UK, an online survey found that 48.2% of parents or guardians would accept COVID-19 vaccination of their children aged 18 months or under [32]. However, in another study conducted in a children’s hospital in Ankara, Turkey, 28.9% of the parents reported that they would not allow their child to be vaccinated with foreign COVID-19 vaccines, while 56.8% said they would if the vaccine was a domestic vaccine [33].

In our survey, the willingness to vaccinate a child for COVID-19 varied between countries and was also found to increase as the vaccine effectiveness level increased. Other studies also showed that the strongest predictor of intent to vaccinate children was vaccine safety and efficacy [19,34,35,36,37]. In a study in the United States, less than one-half of the parents reported that they were likely to have their child receive a COVID-19 vaccine mainly because of safety concerns [38].

The highest vaccination acceptance was noted among the Brazilian participants (95.5%). African and Malaysian participants were less likely to have children vaccinated. It is worth noting that the participants from these countries were significantly younger than Brazilian participants (analysis not shown) and therefore may have had younger children than the other countries. Moreover, at the time of this survey, in Malaysia and Africa, there was a lot of misinformation with regards to the COVID-19 vaccine safety and its efficacy [39,40]. For instance, it was believed that mixed messages coming from political figures created unjustified fears against the adverse effects of H1N1 vaccination [41]. On the other hand, Brazil had the second highest number of COVID-19-related deaths in the world in early 2021 [42]. This situation may have increased the willingness to vaccinate children as the benefits of inoculating against COVID-19 may have been seen as outweighing the potential adverse events.

In our study, male participants were more willing to vaccinate children at the 95% effectiveness level, and this was also seen in a survey in high-income countries [43]. A study on H1N1 vaccination also showed similar results, where fathers were more willing than mothers to vaccinate their child [34]. An explanation could be that males may differ from females in decision-making behavior due to their higher likelihood to take risks. Future in-depth studies are needed to examine these gender dynamics.

Participants from urban areas had 53% lower odds of accepting the vaccine for their children at the 95% effectiveness level. Similarly, past studies also found that caregivers from urban sites were less likely to vaccinate their children than those from rural sites [44]. These results are of concern as the prevalence of COVID-19 positive cases in urban areas has been shown to be higher than in rural areas and small towns [45,46]. Our findings could be due to the greater exposure in urban areas to information from the internet and social media. Online misinformation has been found to correlate with vaccine hesitancy and thus lower the vaccination coverage [19,47,48,49]. Thus, more trusted and reliable sources of information ought to be conveyed to the community, to increase their confidence in the vaccine.

Educational level was strongly associated with willingness to vaccinate children. Undergraduates were more in favor of vaccinating children at all effectiveness levels. Educational level is closely related to knowledge about COVID-19 vaccines. At all three levels of vaccine effectiveness, knowledge about COVID-19 vaccines was associated with willingness to vaccinate a child. So, higher exposure to correct information about COVID-19 benefits vaccine uptake. Knowledge of the disease and understanding that vaccines are effective prevention strategies were also associated with increased H1N1 vaccination uptake [47]. Similar to our findings, parents with post-secondary education have been shown to be more willing to vaccinate their children [44,50,51]. Studies have also shown that children of parents with a higher educational level were more likely to be vaccinated [34,51]. As non-compliance with childhood vaccination has increased in the past ten years [52,53] it is important that decisions to introduce vaccination of children with a new vaccine are taken with sufficient knowledge about the efficacy and potential adverse effects of these vaccines and consider the acceptance of these vaccines by the public, particularly the parents.

Income was also shown to be associated with willingness to vaccinate children against COVID-19. Those with low, lower middle, and upper middle-income status had higher odds of accepting child vaccination at the 95% vaccine effectiveness level, compared to those in the high-income group. These findings contrast with other studies undertaken in the US, where there was a lower level of vaccine acceptance among lower-income groups [54,55]. The COVID-19 pandemic has particularly impacted those belonging to lower socio-economic status groups and thus it is important to prevent further inequity in COVID-19 vaccine distribution and uptake, particularly in disadvantaged communities. Due to the environmental and sanitary conditions that these communities are living in, such as overcrowding, members are more prone to contracting the disease and more likely to die from it, thus, one would expect them to accept the COVID-19 vaccination, not only for their children, but also for themselves.

Participants who tested positive for COVID-19 had 24% lower odds for being prepared to have their children vaccinated at the 50% and 75% vaccine effectiveness levels. The reason for this may be that with such a low level of effectiveness, it may not be viewed as beneficial to expose children to vaccines for which there is limited experience concerning potential side effects in children [56]. Conversely, a previous study found no correlation between family members diagnosed with COVID-19 and their willingness to vaccinate their child [57].

Another factor that may affect the acceptance of the vaccine for children could be living with children, as this may increase the risk of contracting the virus if the children are virus carriers [25]. In our study, those working or studying from home were more open to the vaccination of children. The explanation could be that, for them, contact with children could be their main risk for becoming infected.

Worry about becoming infected with COVID-19 was also significantly associated with vaccine acceptance for children. This concurs with other findings elsewhere. A study found that 92% of caregivers were worried about getting the disease or their family being infected and, among these, 93% would vaccinate their children [31]. Related to the HINI vaccination, there was a strong association between vaccine intentions and fear of the adult and child catching the disease [19], while concern about the COVID-19 outbreak in Australia was associated with enhanced willingness to get vaccinated [58]. According to the health belief model, those who were more worried/fearful of COVID-19 may be more likely to seek relief for their adverse emotional condition by accepting the vaccine, including for children [59]. Therefore, fear or worry could be a catalyst for the vaccination of children and to achieve herd immunity. For the uptake of health behaviors, an understanding of these factors affecting people’s intentions for vaccination is deemed important for the success of any vaccination program. The factors highlighted in this study will further assist policy makers to plan and develop strategies for their target groups for vaccinations, based on socio-demographic data.

In our study, those who believed that vaccination was important to protect others had 2.33 times, 1.58 times and 1.61 times higher odds of accepting having children vaccinated at 95%, 75%, and 50% vaccine effectiveness, respectively. Similarly, a study found that the main reasons for vaccine acceptance was self-protection (42%) and protecting the child (42%) from COVID-19, with 48.2% leaning towards accepting a COVID-19 vaccine for their child [32]. Goldman and colleagues reported that the most common reason given by caregivers intending to vaccinate their children was to protect their children (62%) [20]. Similar to our findings, Goldman and colleagues reported that “protection of others” was the second common theme given by their willing caregivers. The low prevalence of acceptance could be because children have a lower susceptibility to develop severe COVID-19 disease and their role in the transmission of COVID-19 is unclear [60,61].

A recent review about the reasons for vaccine hesitancy in LMICs showed that exposure to misinformation about COVID-19 vaccines, public concerns over the safety of vaccines, and distrust in the government, were the main contributing factors to low vaccine acceptance rates [18]. Therefore, to decrease vaccine hesitancy, direct engagement with communities through influencers, including community leaders and health experts, is needed. Moreover, clear and transparent communication, with strong endorsement by health care workers, is very important [62]. Thus, clear and consistent communication and accurate information is important in helping people decide whether they want to be vaccinated or not. Further, whether it is vaccines for adults or children, policy makers, government officials, and the media should pay attention to the spread of data which is not supported by scientific evidence that may affect vaccine acceptance [62].

Our study is not without limitations. Our method of using online social media and online communicators, such as WhatsApp and Facebook, to distribute the questionnaire could lead to biased results. This is because individuals belonging to low-income countries may have limited internet access, and therefore the responses may originate from individuals with higher income levels who could afford an electronic device or internet connection to access this questionnaire. Moreover, it may also limit the participants to those within the researchers’ personal and professional networks, and this creates a non-representative, biased study population. Future studies should consider conducting surveys selecting participants randomly via telephone or a paper questionnaire to ensure a more representative study population. However, such surveys are more difficult to organize, are not feasible in certain settings (telephone numbers may not be available) and are costly. Due to the COVID-19 pandemic, we have chosen the online survey method to obtain rapid information at low cost as a basis for designing more in-depth studies when the COVID-19 vaccines will be more widely available for children. We considered the household composition of the participants, but we did not specifically ask parents whether they would be willing to vaccinate their own children. We also did not specify the child’s age at which they would accept vaccination—the decision to vaccinate a young child may be seen as being riskier than to vaccinate a near-adult child. Other influencing factors also need to be considered, such as attitudes towards all vaccinations, and social media influence which can affect ones’ perception of vaccine acceptability. The validity, reliability, and intelligibility of the questionnaire was not thoroughly assessed in the target population. We did not specify in the questionnaire the meaning of the term “effectiveness of the COVID-19 vaccine”; this could be effectiveness against infection, severe COVID-19, hospitalization, or death. It seems most probable that most respondents interpreted the question as effectiveness against infection. As in any cross-sectional study design, causal inference cannot be made. It is also important to mention that this survey was carried out prior to the COVID-19 vaccination roll-out in the surveyed countries in this study. So, over time, perceptions about COVID-19 vaccination may have changed. For example, studies investigating the safety, immunogenicity, and efficacy of the COVID-19 vaccine in children [63] may change parents’ and caregivers’ willingness to vaccinate their children.

In November 2021, the US Centers for Disease Control and Prevention recommended Pfizer’s COVID-19 mRNA vaccine for children between 5 and 11 years [64]. Yet surveys in the United States showed that 42% to 66% of parents were reluctant or opposed to vaccinate their children [64]. Without vaccination, it is likely that almost everyone, including young children, will be COVID-19 infected at some point in their lives [65]. So, the question is: which is worse for children, vaccination or natural infection? In high-income countries, vaccination of children has started on a large scale and vaccine acceptance rates have also changed over time [65]. The successful vaccination program in Israel, and to a lesser degree in the United States, led to increased willingness by parents to vaccinate their children younger than 12 years against COVID-19. In Canada, a low rate of vaccination of adults was associated with lower willingness to vaccinate children [65]. A follow-up study should also be carried in LMICs once vaccination for children against COVID-19 has become more widely available.

## 5. Conclusions

More than half the respondents were willing to vaccinate children. Factors influencing willingness to vaccinate children were parental gender, age, education, income level, residential setting, knowledge, worry about being infected, and the importance of vaccination to protect self or others. Findings from this study are useful for policy decisions about COVID-19 vaccination of children. Vaccination may reduce the spread of infection in schools and nurseries. However, according to current evidence, children rarely develop severe symptoms due to COVID-19; therefore, administering the COVID-19 vaccine in children may be of limited or no benefit for the health of the children. Alternatively, vaccinating school children may decrease COVID-19 transmission in school and in this way may avoid school closures. Keeping schools open is very important for the education and development of the children. Another reason to vaccinate children, still being assessed, is to prevent them transmitting the infection to persons at risk of severe disease, such as elderly people and persons with underlying chronic diseases. However, given that the latter are increasingly being protected by vaccination, and other public health interventions, the need to vaccinate children, and at what age, are still very much under debate. Within this debate, the opinion of the children and their parents needs to be considered to design appropriate policies regarding vaccination of children against COVID-19.

## Figures and Tables

**Table 1:** Participants’ demographics and health status (*n* = 6571).

Variable	Total	Brazil *n* = 4867	Malaysia *n* = 1245	Thailand *n* = 122	Bangladesh *n* = 199	African Countries ^†^ *n* = 138
	*n* (%)	*n* (%)	*n* (%)	*n* (%)	*n* (%)	*n* (%)
Demographics						
Gender						
Male	2312 (35.2)	1661 (34.1)	446.0 (35.8)	32.0 (26.2)	79.0 (39.7)	94.0 (68.1)
Female	4259 (64.8)	3206 (65.9)	799.0 (64.2)	90.0 (73.8)	120.0 (60.3)	44.0 (31.9)
Age, years						
Mean ± SD	47 ± 15	49 ± 14	41 ± 15	47 ± 10	29 ± 6	35 ± 9
Median (Min, Max)	48 (18, 93)	51 (18, 93)	38 (18, 82)	47 (24, 76)	29 (18, 60)	35 (18, 65)
18–29	945 (14.4)	414 (8.5)	357 (28.7)	7 (5.7)	119 (59.8)	48 (34.8)
30–39	1320 (20.1)	895 (18.4)	286 (23.0)	24 (19.7)	72 (36.2)	43 (31.2)
40–49	1254 (19.1)	1001 (20.6)	172 (13.8)	38 (31.1)	5 (2.5)	38 (27.5)
50–59	1498 (22.8)	1239 (25.5)	206 (16.5)	45 (36.9)	1 (0.5)	7 (5.1)
60 and above	1554 (23.6)	1318 (27.1)	224 (18.0)	8 (6.6)	2 (1.0)	2 (1.4)
Education level						
Primary/Secondary	841 (12.8)	580 (11.9)	230 (18.5)	6 (4.9)	17 (8.5)	8 (5.8)
Undergraduate Degree	2425 (36.9)	1508 (31.0)	652 (52.4)	63 (51.6)	109 (54.8)	93 (67.4)
Postgraduate Degree	3305 (50.3)	2779 (57.1)	363 (29.2)	53 (43.4)	73 (36.7)	37 (26.8)
Socio-economic status						
Low/Lower Middle	3177 (48.3)	2206 (45.3)	694 (55.7)	92 (75.4)	82 (41.2)	103 (74.6)
Upper Middle	3025 (46.0)	2342 (48.1)	512 (41.1)	26 (21.3)	111 (55.8)	34 (24.6)
High	369 (5.6)	319 (6.6)	39 (3.1)	4 (3.3)	6 (3.0)	1 (0.7)
Residential setting						
Rural	294 (4.5)	111 (2.3)	155 (12.4)	23 (18.9)	3 (1.5)	2 (1.4)
Suburban/Slum	786 (12.0)	542 (11.1)	182 (14.6)	19 (15.6)	29 (14.6)	14 (10.1)
Urban	5491 (83.6)	4214 (86.6)	908 (72.9)	80 (65.6)	167 (83.9)	122 (88.4)
Healthcare worker or student (Yes)	1968 (29.9)	1443 (29.6)	270 (21.7)	49 (40.2)	116 (58.3)	90 (65.2)
Working/studying from home (Yes)	3835 (58.4)	2885 (59.3)	729 (58.6)	50 (41.0)	125 (62.8)	46 (33.3)
COVID-19 testing						
Not tested/Don’t know test results	3614 (55.0)	2444 (50.2)	873 (70.1)	103 (84.4)	111 (55.8)	83 (60.1)
Tested, negative	2379 (36.2)	1893 (38.9)	360 (28.9)	19 (15.6)	56 (28.1)	51 (37.0)
Tested, positive	578 (8.8)	530 (10.9)	12 (1.0)	0 (0.0)	32 (16.1)	4 (2.9)
Household						
Living with children < 12 years old	2004 (30.5)	1417 (29.1)	395 (31.7)	25 (20.5)	81 (40.7)	86 (62.3)
Living with children 12 to 17 years old	1350 (20.5)	902 (18.5)	312 (25.1)	21 (17.2)	47 (23.6)	68 (49.3)

^†^ Countries in Africa in this study comprised of DR Congo (*n* = 117), Uganda (*n* = 8), and Malawi (*n* = 13).

**Table 2:** Knowledge and attitudes towards COVID-19 vaccination.

Variable	Total	Brazil *n* = 4867	Malaysia *n* = 1245	Thailand *n* = 122	Bangladesh *n* = 199	African Countries ^†^ *n* = 138
	*n* (%)	*n* (%)	*n* (%)	*n* (%)	*n* (%)	*n* (%)
Willingness to vaccinate children						
At 50% Effectiveness	3691 (64.0)	3313 (77.1)	289 (27.2)	15 (13.0)	59 (34.1)	15 (12.4)
At 75% Effectiveness	4432 (72.6)	3917 (85.2)	383 (35.2)	23 (20.2)	93 (50.0)	16 (13.4)
At 95% Effectiveness	6107 (92.9)	4667 (95.9)	1060 (85.1)	109 (89.3)	185 (93.0)	86 (62.3)
Worry/fear of being infected with COVID-19 (Likert score, 1–5)						
Mean ± SD	3.63 ± 1.09	3.75 ± 1.05	3.52 ± 1.04	3.02 ± 1.10	2.76 ± 1.01	2.35 ± 1.10
Median (Min, Max)	4 (1,5)	4.00 (1,5)	4.00 (1,5)	3.00 (1,5)	3 (1,5)	2.00 (1,5)
Knowledge about COVID-19 (Yes): *n* (%)						
Possibility of being re-infected after recovering from COVID-19	5514 (83.9)	4180 (85.9)	956 (76.8)	98 (80.3)	177 (88.9)	103 (74.6)
COVID-19 can be prevented by vaccination	5407 (82.3)	4428 (91.0)	715 (57.4)	78 (63.9)	108 (54.3)	78 (56.5)
There is currently an effective vaccine against COVID-19	4361 (66.4)	3738 (76.8)	421 (33.8)	80 (65.6)	88 (44.2)	34 (24.6)
Knowledge about COVID-19 (composite score, 0–3) *						
Mean ± SD	2.33 ± 0.88	2.54 ± 0.75	1.68 ± 0.93	2.10 ± 0.86	1.87 ± 0.90	1.56 ± 0.94
Median (Min, Max)	3 (0, 3)	3 (0, 3)	2 (0, 3)	2 (0, 3)	2 (0, 3)	1.5 (0, 3)
Importance of COVID-19 vaccination to protect others (Likert score, 1–5)						
Mean ± SD	4.87 ± 0.85	4.76 ± 0.65	4.09 ± 1.04	3.75 ± 1.00	4.17 ± 0.88	3.36 ± 1.49
Median (Min, Max)	5 (1,5)	5 (1,5)	4 (1,5)	4 (1,5)	4 (1,5)	4 (1,5)
Not at all important	158 (2.4)	73 (1.5)	45 (3.6)	6 (4.9)	3 (1.5)	31 (22.5)
A little important	97 (1.5)	28 (0.6)	57 (4.6)	1 (0.8)	3 (1.5)	8 (5.8)
Moderately important	326 (5.0)	73 (1.5)	177 (14.2)	26 (21.3)	36 (18.1)	14 (10.1)
Very important	1258 (19.1)	651 (13.4)	433 (34.8)	51 (41.8)	73 (36.7)	50 (36.2)
Extremely important	4732 (72.0)	4042 (83.0)	533 (42.8)	38 (31.1)	84 (42.2)	35 (25.4)

Note. Percentages (%) are within country comparisons. ^†^ Countries in Africa in this study comprised of DR Congo (*n* = 117), Uganda (*n* = 8), and Malawi (*n* = 13). * Reference Group.

**Table 3:** Bivariate analysis of associations between demographic variables, knowledge, and attitudes towards COVID-19 vaccination and vaccine effectiveness.

Variable	50% Effectiveness				75% Effectiveness				95% Effectiveness			
	*n* (%)	χ^2^	*p*	Effect Size *	*n* (%)	χ^2^	*p*	Effect Size *	*n* (%)	χ^2^	*p*	Effect Size *
Age (Years)		152.96	<0.001	0.163		138.97	<0.001	0.151		18.21	0.001	0.053
18–29	401 (50.5)				507 (59.9)				859 (90.9)			
30–39	656 (57.4)				827 (68.1)				1226 (92.9)			
40–49	683 (62.6)				841 (71.8)				1148 (91.5)			
50–59	925 (68.8)				1078 (76.4)				1402 (93.6)			
60 and above	1026 (73.4)				1179 (80.6)				1472 (94.7)			
Country		1277.05	<0.001	0.470		1545.75	<0.001	0.503		379.54	<0.001	0.240
Brazil	3313 (77.1)				3917 (85.2)				4667 (95.9)			
Malaysia	289 (27.2)				383 (35.2)				1060 (85.1)			
Thailand	15 (13.0)				23 (20.2)				109 (89.3)			
Bangladesh	59 (34.1)				93 (50.0)				185 (93.0)			
African Countries ^†^	15 (12.4)				16 (13.4)				86 (62.3)			
Gender		12.43	<0.001	0.046		20.58	<0.001	0.058		19.46	<0.001	0.054
Male	1264 (61.0)				1509 (69.1)				2105 (91.0)			
Female	2427 (65.6)				2923 (74.5)				4002 (94.0)			
Education Level		109.43	<0.001	0.138		137.42	<0.001	0.150		61.39	<0.001	0.097
Primary/Secondary	398 (55.4)				486 (64.3)				748 (88.9)			
Undergraduate	1220 (57.9)				1466 (66.2)				2209 (91.1)			
Postgraduate	2073 (70.4)				2480 (79.1)				3150 (95.3)			
Household												
Living with children <12 years old		47.68	<0.001	0.091		50.58	<0.001	0.091		11.55	0.001	0.042
Yes	989 (57.3)				1210 (66.4)				1830 (91.3)			
No	2702 (66.8)				3222 (75.2)				4277 (93.7)			
Living with children 12 to 17 years old		22.76	<0.001	0.063		30.14	<0.001	0.070		13.36	<0.001	0.045
Yes	678 (58.0)				819 (66.4)				1224 (90.7)			
No	3013 (65.5)				3613 (74.2)				4883 (93.5)			
Socio-economic status		49.68	<0.001	0.093		80.18	<0.001	0.115		16.22	<0.001	0.050
Low/Lower Middle	1669 (59.9)				1971 (67.4)				2911 (91.6)			
Upper Middle	1770 (66.7)				2168 (76.7)				2850 (94.2)			
High	252 (76.1)				293 (82.3)				346 (93.8)			
Residential setting		76.99	<0.001	0.116		88.80	<0.001	0.121		3.49	0.175	0.023
Suburban/Urban Slum	433 (62.1)				515 (70.0)				726 (92.4)			
Urban	3166 (65.5)				3796 (74.2)				5115 (93.2)			
Rural	92 (38.0)				121 (47.6)				266 (90.5)			
Healthcare worker or student		5.65	0.017	0.031		3.61	0.057	0.024		0.39	0.530	0.008
Yes	1076 (61.7)				1296 (70.9)				1835 (93.2)			
No	2615 (65.0)				3136 (73.3)				4272 (92.8)			
Working/studying from home		24.18	<0.001	0.065		45.53	<0.001	0.086		19.15	<0.001	0.054
Yes	2221 (66.6)				2709 (75.8)				3609 (94.1)			
No	1470 (60.3)				1723 (68.0)				2498 (91.3)			
COVID-19 Testing		20.79	<0.001	0.060		21.73	<0.001	0.060		8.03	0.018	0.035
Tested Negative	1382 (66.1)				1645 (74.4)				2237 (94.0)			
Tested Positive	364 (70.3)				431 (78.6)				540 (93.4)			
Not Tested/ Does not know test results	1945 (61.6)				2356 (70.4)				3330 (92.1)			
	Mean (SD)	*t* value	*p*	Effect size §	Mean (SD)	*t* value	*p*	Effect size §	Mean (SD)	*t* value	*p*	Effect size §
Worry/fear of being infected with COVID-19	3.86 (0.98)	20.13	<0.001	0.694	3.82 (0.98)	19.29	<0.001	0.766	3.71 (1.03)	16.65	<0.001	1.479
Knowledge about COVID-19 ‡	2.65 (0.62)	35.88	<0.001	1.308	2.63 (0.63)	39.73	<0.001	1.678	2.41 (0.81)	30.57	<0.001	0.754
Importance of vaccination to protect others	4.84 (0.43)	28.03	<0.001	1.148	4.82 (0.44)	28.93	<0.001	1.352	4.69 (0.61)	24.78	<0.001	2.276

Note. ^†^ Countries in Africa in this study comprised of DR Congo (*n* = 117), Uganda (*n* = 8), and Malawi (*n* = 13). ‡ Knowledge was the sum of answering correctly (= 1 point each) to the following questions: (1) possibility of being re-infected after recovering from a previous COVID-19 infection; (2) COVID-19 infection could be prevented by a vaccine; and (3) there is currently an effective vaccine against COVID-19. * Cramer’s V. § Cohen’s d.

**Table 4:** Multiple logistic regression models of factors predicting vaccine acceptance for child at 50%, 75%, and 95% effectiveness levels.

Variables	50% Effectiveness ^a^				75% Effectiveness ^b^				95% Effectiveness ^c^			
	aOR	95% CI		*p*-Value	aOR	95% CI		*p*-Value	aOR	95% CI		*p*-Value
		Lower	Upper			Lower	Upper			Lower	Upper	
Constant	0.05				0.09				0.05			
Age (Years)												
18–29	0.62	0.46	0.82	0.001	0.63	0.46	0.86	0.003	0.98	0.64	1.49	0.922
30–39	0.47	0.36	0.62	<0.001	0.47	0.35	0.63	<0.001	2.00	1.31	3.05	0.001
40–49	0.85	0.64	1.13	0.253	0.73	0.53	0.99	0.040	1.17	0.77	1.78	0.466
50–59	0.73	0.56	0.96	0.025	0.66	0.49	0.89	0.006	1.78	1.14	2.77	0.011
60 and above *												
Country												
Malaysia	0.18	0.13	0.24	<0.001	0.16	0.12	0.22	<0.001	0.58	0.38	0.88	0.011
Thailand	0.06	0.05	0.08	<0.001	0.06	0.04	0.08	<0.001	0.78	0.53	1.15	0.217
Bangladesh	0.34	0.27	0.42	<0.001	0.37	0.29	0.46	<0.001	1.46	1.00	2.14	0.051
African Countries ^†^	0.11	0.09	0.15	<0.001	0.07	0.05	0.10	<0.001	0.23	0.16	0.32	<0.001
Brazil *												
Gender (Female, ref Male)	1.03	0.89	1.19	0.668	1.17	1.01	1.35	0.032	0.55	0.45	0.68	<0.001
Education Level												
Primary/Secondary *												
Undergraduate	1.28	1.00	1.64	0.048	1.38	1.08	1.77	0.010	2.33	1.71	3.19	<0.001
Postgraduate	0.81	0.63	1.04	0.100	0.93	0.72	1.20	0.577	1.84	1.31	2.57	<0.001
Household												
Living with children < 12 years old (Yes)	0.90	0.76	1.05	0.178	1.01	0.86	1.19	0.873	1.30	1.04	1.63	0.023
Living with children 12 to 17 years old (Yes)	1.27	1.07	1.50	0.006	1.14	0.96	1.34	0.132	0.88	0.71	1.11	0.281
No *												
Socio-economic status												
Low/Lower Middle	1.12	0.78	1.60	0.534	0.78	0.53	1.15	0.211	2.34	1.37	3.98	0.002
Upper Middle	1.02	0.71	1.44	0.933	0.87	0.60	1.28	0.484	1.77	1.04	3.00	0.034
High *												
Residential setting												
Suburban/Urban Slum	1.20	0.80	1.80	0.379	1.28	0.87	1.86	0.207	0.69	0.39	1.24	0.220
Urban	1.23	0.85	1.78	0.274	1.37	0.97	1.93	0.070	0.47	0.28	0.79	0.005
Rural *												
Healthcare worker or student (Yes, reference group = No)	0.98	0.85	1.14	0.830	0.94	0.81	1.10	0.441	0.96	0.78	1.17	0.674
Working/studying from home (Yes, reference group = No)	1.36	1.18	1.56	<0.001	1.21	1.05	1.39	0.010	0.94	0.77	1.14	0.526
COVID-19 Testing												
Tested Negative	0.88	0.76	1.03	0.121	0.70	0.60	0.82	<0.001	1.23	0.98	1.53	0.070
Tested Positive	0.78	0.61	0.99	0.043	0.76	0.60	0.97	0.027	1.38	0.93	2.04	0.111
Not Tested/ Does not know test results *												
Worry/fear of being infected with COVID-19	1.17	1.09	1.25	<0.001	1.08	1.01	1.16	0.018	1.31	1.19	1.43	<0.001
Knowledge about COVID-19 ^‡^	1.79	1.65	1.95	<0.001	2.17	1.99	2.36	<0.001	1.66	1.48	1.86	<0.001
Importance of vaccination to protect others	1.61	1.47	1.76	<0.001	1.58	1.45	1.72	<0.001	2.33	2.14	2.53	<0.001

Note. All predictors listed in this table were entered into the regression models with 50%, 75%, and 95% effectiveness levels as the criterion variables. ^a^ χ^2^(24) = 2562.76, *p* < 0.001; Nagelkerke R^2^ = 0.48. ^b^ χ^2^(24) = 3042.69, *p* < 0.001; Nagelkerke R^2^ = 0.53. ^c^ χ^2^(24) = 2019.51, *p* < 0.001; Nagelkerke R^2^ = 0.49. * Reference Group. ^†^ Countries in Africa in this study comprised of DR Congo, Uganda, and Malawi, ^‡^ Knowledge was the sum of answering correctly (=1 point each) to the following questions: (1) possibility of being re-infected after recovering from a previous COVID-19 infection; (2) COVID-19 infection could be prevented by a vaccine; and (3) there is currently an effective vaccine against COVID-19.

## Data Availability

Data are available upon reasonable request. Data are available on the International Consortium (International Citizen Project COVID-19 (ICPcovid): http://www.icpcovid.com, accessed on 4 November 2021) website and can be used by other investigators on request. De-identified participant data are available.
